# Exploring Cardiovascular Manifestations in Vasculitides: An In-Depth Review

**DOI:** 10.7759/cureus.44417

**Published:** 2023-08-30

**Authors:** Vaibhav Vats, Kriyesha Patel, Dhruvikumari D Sharma, Naiela E Almansouri, Naga Sai Ram Makkapati, Simran Nimal, Palash Ramteke, Bushra Mohammed Arifuddin, Nikhil Sai Jagarlamudi, Archit Narain, Yogesh D Raut

**Affiliations:** 1 Internal Medicine, Smt. Kashibai Navale Medical College and General Hospital, Mumbai, IND; 2 Internal Medicine, MP Shah Medical College, Jamnagar, IND; 3 Internal Medicine, Spartan Health Sciences University, Vieuxfort, LCA; 4 Internal Medicine, University of Tripoli, Tripoli, LBY; 5 Internal Medicine, NRI Medical College, Vijayawada, IND; 6 Internal Medicine, Byramjee Jeejeebhoy (BJ) Government Medical College, Pune, IND; 7 Medical School, NKP Salve Institute of Medical Sciences, Nagpur, IND; 8 Internal Medicine, Shadan Institute of Medical Sciences and Teaching Center, Hyderabad, IND; 9 Internal Medicine, NRI Medical college, Guntur, IND; 10 Internal Medicine, Lala Lajpat Rai Memorial Medical College, Meerut, IND; 11 Miscellaneous, NKP Salve Institute of Medical Sciences, Nagpur, IND

**Keywords:** myocarditis, vasculitides, myocardial infarction, takayasu arteritis, kawasaki disease, giant cell arteritis, cardiovascular, vasculitis

## Abstract

Systemic vasculitides encompass a cluster of autoimmune diseases that affect blood vessels, and are characterized by immune-mediated injury to either small- or large-sized blood vessels. Individuals afflicted with systemic vasculitides experience notable morbidity and mortality attributable to cardiovascular manifestations. Noteworthy among these are ischemic heart disease, venous thromboembolism, aortic involvement, valvular irregularities, myocarditis, and pericarditis. This narrative review investigated and evaluated the prevalent cardiovascular disturbances commonly associated with different types of vasculitides. This review also discusses the mechanisms that underlie these manifestations. It also provides a thorough explanation of the many diagnostic techniques essential for detecting the disease at its occult stage. It is essential for healthcare professionals to have knowledge of the cardiovascular complications caused by vasculitides, as this enables them to promptly recognize these symptoms and employ suitable diagnostic techniques early on. By doing so, timely detection can be ensured, which will subsequently aid in initiating appropriate treatment strategies that are vital for decreasing morbidity and mortality in patients with systemic vasculitides.

## Introduction and background

Systemic vasculitides encompass a cluster of autoimmune pathologies that affect blood vessels marked by immune-mediated injury affecting small to large blood vessels, involving either single or multiple organ systems [1]. With every million individuals in the general population, 40-60 cases are diagnosed every year [2].

Apart from categorization based on the diameter of the involved vessels, vasculitides can either be a primary pathology or sequelae to preceding connective tissue disorders or secondary insults like specific drugs [3]. Vasculitides, which are associated with endothelial activation and damage, have a substantial impact on the cardiovascular system [4].

Involvement of the major coronary and cerebral arteries has led to a high number of debilitating cardiovascular morbidities [5]. A notably high prevalence of secondary hypertension and dyslipidemia has been noticed in individuals with established systemic disease and can act as possible predecessors of angina, ischemic heart diseases, strokes, peripheral vascular diseases, and aortic aneurysms in this group [6]. Long-standing vascular inflammation seen in these patients has also been associated with higher incidences of pericarditis, cardiomyopathies, myocardial ischemia, and fibrosis, which also affect cardiac contractility and lead to heart failure [7].

Patients with a confirmed diagnosis may undergo non-invasive screening procedures, such as electrocardiography and transthoracic echocardiography, to evaluate the presence of any cardiovascular pathology [8]. Fluorodeoxyglucose (FDG)-positron emission tomography (PET) and cardiac magnetic resonance imaging (MRI) are two more cutting-edge diagnostic technologies that may be used for the early detection of any small abnormalities that might be missed with traditional screenings [9].

While the impact of vasculitides on various organ systems has been extensively explored, there exists a gap in comprehensively addressing cardiovascular manifestations, their underlying pathophysiology, and the clinical implications they carry. Our review aimed to address this gap by providing an in-depth account of the cardiovascular manifestations associated with different types of vasculitides, elucidating their underlying mechanisms, and highlighting the role of various diagnostic modalities that help detect the disease at an occult stage, thus facilitating prompt interventions to mitigate potential long-term cardiac repercussions.

## Review

Takayasu arteritis

Takayasu arteritis (TA), referred to as a pulseless disease, is an inflammatory condition that results in immune-driven harm to the aorta and its primary vessels, notably to the renal, carotid, and subclavian arteries. This in turn results in narrowing, blockage, or abnormal dilation of these arteries due to inflammation [10]. Pan-luminal inflammation initiates a remodeling process that leads to the development of aneurysms and lumen occlusive intimal hyperplasia [11]. It predominantly impacts young Asian women between the ages of 18 and 40 years, with a female-to-male ratio of 9:1 [10].

Approximately 90% of individuals diagnosed with TA display varying degrees of aortic involvement, with 30-60% progressing to ascending aortic regurgitation [12]. Most patients also suffer from subclavian stenosis, causing diminished or no pulses in the arm [11]. Other locations, such as the carotid arteries, which can cause cerebral ischemia; the renal arteries, which can result in hypertension; and the pulmonary arteries, which can cause coughing, shortness of breath, chest pain, and pulmonary hypertension in patients, can also be involved [13]. Younger patients are more likely to experience major ischemic events, including myocardial infarction (MI), stroke, renal artery involvement, and constitutional symptoms associated with active illness [14].

Patients with TA frequently have valvular insufficiency, with aortic regurgitation being the most common. Ren et al. found that out of 103 TA patients in their study, 64% had valvular involvement, 62% had aortic regurgitation, 60.61% had mitral valve involvement, 54.6% had tricuspid valve involvement, and 6.1% had pulmonary insufficiency [15]. Zhang et al. found that out of 1069 patients, 34.9% had regurgitation, of which 69.7% had aortic regurgitation [16]. Mitral valve failure accounted for 39.1%, tricuspid valve insufficiency for 34.2%, and pulmonary regurgitation for 11.8% of the patients [16].

Valvular involvement in patients with TA can lead to myocardial remodeling, left ventricular dysfunction, and increased afterload, culminating in heart failure - the primary contributor to mortality among individuals with TA [17]. Color Doppler echocardiography is more sensitive for the diagnosis of valvular disorders than angiographic evaluation in TA, thereby improving the incidence rate [18].

Prednisolone is the first-line therapy for TA and helps prevent the onset of vascular and valvular pathologies, while adjunctive steroid-sparing immunosuppressants like methotrexate and azathioprine are crucial for remission and halting arterial lesions’ progression in patients intolerant to steroid treatment because of its side effects [19]. TNF-α antagonists and anti-IL6 receptor monoclonal antibodies have been shown to induce remission and treat refractory diseases, respectively, in a few patients [19]. Revascularization surgery is required for symptomatic stenotic and occlusive lesions. Stenosis, severe regurgitation, occlusive lesions, and the possibility of aneurysm rupture are indications for surgical intervention [19].

Coronary arterial involvement is limited in patients with TA [20]. Inflammation-induced intimal proliferation and subsequent fibrosing contraction underlie coronary involvement [21]. Of the 130 TA patients studied by Endo et al., 31 had abnormal coronary angiographic findings, including 24 with coronary artery stenosis (greater than 75%) [20]. Ci et al. discovered 207 coronary lesions in 87 TA patients, with men having more severe coronary lesions than women (p=0.031) and a reduced prevalence of lesions of coronary ostia [22]. Furthermore, mortality was higher in males than in women, suggesting a greater likelihood of significant coronary narrowing and increased long-term mortality risk in males with coronary artery involvement [22]. MRI has been suggested as the primary imaging method for cases of suspected TA [23]. Ultrasound is particularly useful in the detection of early pre-stenotic arterial wall lesions [24], along with MR-angiography, which is contrast-dependent but without the risk of radiation and hence useful in younger TA patients [25]. The initial treatment of choice for coronary artery anomalies in TA patients is prednisone (40-60 mg/day) [26]. During the early phases of the condition, percutaneous coronary intervention (PCI) demonstrates outcomes comparable to those of cardiac bypass surgery (CABG), with the latter being more suitable for individuals with ongoing active disease necessitating urgent revascularization [27].

According to a study conducted by Fateh-Moghadam et al., only four instances of pericardial effusion associated with TA have been documented thus far [28]. Although angiography has traditionally been the conventional diagnostic approach, there has been a shift towards adopting less invasive techniques such as CT angiography and magnetic resonance angiography (MRA). These alternatives enable the evaluation of wall thickness and help prevent false-negative results during the initial phases of the assessment [29]. Acute pericarditis is treated with NSAIDs and colchicine, with corticosteroids as second-line treatment reserved for unresponsive cases, although relapses are frequent [30].

Myocarditis-related cardiac dysfunction (inflammatory cardiomyopathy) is not commonly described; they are typically discussed in autopsy studies [31,32]. Bechman et al. studied 139 TA patients and 24 giant cell arteritis (GCA) patients and found 2.8% of cases with myocarditis [33]. Transthoracic echocardiography and cardiac magnetic resonance (CMR) are non-invasive methods capable of effectively identifying clinically significant myocarditis, offering a viable substitute for the established benchmark of myocardial biopsy [33]. Kotake et al. showed that steroids, immunosuppressants, and traditional heart failure therapies lead to an improvement and reduction in left ventricular dysfunction and myocarditis [34].

Although relatively uncommon, myocardial ischemia stands out as a major causative factor of fatalities in TA, resulting in mortality rates reaching 50% within a five-year span [35]. In the active phase of TA, impaired endothelial function, platelet aggregation, and heightened coagulation activity collectively contribute to acute myocardial infarction [36]. Endovascular procedures such as percutaneous transluminal angioplasty and stent placement are viable options once the patient's state has stabilized during the remission phase. If attempted earlier, these interventions can potentially exacerbate the vascular wall [37].

Giant cell arteritis

The primary vasculitis most common in the Western hemisphere is giant cell arteritis (GCA), which mostly affects people over the age of 50 and is most prevalent in people between the ages of 70 and 80 [38]. The temporal artery and extracranial branches of the external carotid artery are the most commonly affected arteries in the GCA [39]. Approximately 27-56% of GCA cases share association and pathogenic roots with polymyalgia rheumatica (PMR) [40].

Although not fully understood, the inclination to develop GCA is thought to result from a combination of various genetic and environmental factors. These factors stimulate the dendritic cells (DC) within the tunica adventitia, subsequently triggering a T-cell response [41]. Over time, chronic inflammation leads to damage and remodeling of the arterial walls, culminating in progressive lumen narrowing, which is responsible for ischemic complications of the disease [42]. Temporal artery biopsy is the preferred diagnostic approach, which reveals the characteristic presence of multinucleated giant cells in over 50 percent of the samples [41].

In numerous studies, GCA has been associated with various cardiovascular morbidities, as observed in a retrospective cohort in Canada [43] and a UK-based cohort analysis [44]. Amiri et al. found a three-fold increased risk of MI in GCA cohorts with the risk being highest in the 1st year (adjusted HR = 1.77; 95% CI 1.29-2.43 & 1st year HR = 4.76; 95% CI 3.29-6.88) [45]. Evans et al. found that patients with GCA had a 17.3 times higher risk (95% CI 7.9 - 33) of developing thoracic aortic aneurysm and a 2.4 times higher risk (CI 0.8 - 5.5) of developing solitary abdominal aortic aneurysm [46]. In a cohort from the same population, Nuenninghoff et al. identified 27% of GCA cases with large-artery complications, of which 18% were aortic pathologies (dissection or aneurysm) [47]. Compared to matched controls, Li et al. found that GCA patients had a higher risk of vascular disorders [48]. These patients exhibited a higher incidence of myocardial infarction (HR=1.57; 95% CI 1.36-1.82), peripheral vascular disease (HR=1.75; 95% CI 1.49-2.06), venous thromboembolism (HR=2.03; 95% CI 1.77-2.33), stroke (HR=1.75; 95% CI 1.49-2.06) as well as aortic aneurysm (HR=1.98; 95% CI 1.50-2.62) [48]. GCA occasionally presents as myocarditis [49] and pericarditis [50].

The first-line treatment for GCA is glucocorticoids, which control inflammation and reduce the risk of blindness [51]. Patients with a high likelihood of positive results can be initiated on glucocorticoid therapy before confirmation of the disease with a biopsy [52]. Stone et al. found that the IL-6 inhibitor tocilizumab has much higher rates (56% and 53%) of persistent remission than prednisone [53].

Kawasaki disease

Kawasaki disease predominantly affects children under the age of five. The main signs of this disease include fever, bilateral conjunctival congestion, cracked lips, and erythematous oral and pharyngeal mucosa [54]. Various cardiovascular complications such as endocarditis, myocarditis, valvular involvement, and coronary artery disease play a significant role in long-term health complications and mortality in patients with Kawasaki disease [55]. In a study conducted by Harada et al., they examined 29 Kawasaki disease (KD) patients who died within 40 days of the onset of their illness [56]. The results confirmed that coronary arteritis was present in 27 of the 29 patients that were studied. Specifically, an aneurysm in the left anterior descending coronary artery was found in 24 individuals, with thrombotic closure occurring in 16 cases. Additionally, aneurysms were observed in the left circumflex and right coronary arteries, affecting 16 and 17 patients, respectively, [56]. Myocarditis is another common finding in the early stages of KD. Although many studies have indicated that treatment can improve cardiac function, indications of lasting myocardial abnormalities have also been observed [57]. Tsuda et al. conducted nationwide surveys in Japan from 2007 to 2016, focusing on 137,026 patients with Kawasaki disease [58]. One month after the onset of Kawasaki disease, only 290 (0.2%) patients showed valvular sequelae, 183 (63%) had mitral regurgitation (MR), 112 (39%) had tricuspid regurgitation, 39 (13%) had aortic regurgitation, and 49 (17%) had pulmonary regurgitation [58]. Echocardiography is the principal imaging method for cardiac assessment in KD patients [59]. The primary treatment for Kawasaki disease consists of a combination of acetylsalicylic acid (ASA) and intravenous immunoglobulin (IVIG) [55].

Polyarteritis nodosa

Polyarteritis nodosa (PAN) is a form of systemic vasculitis characterized by necrosis of the blood vessel walls, with a predilection for medium-sized arteries. While viral infections, particularly hepatitis B virus infections, can cause PAN, the condition is mostly idiopathic [60]. In patients with PAN, general symptoms include fever, weight loss, myalgia, arthralgia, peripheral neuropathy, hematuria, proteinuria, recent-onset hypertension, nodules, purpura, etc [61]. The cardiovascular manifestations of PAN include vasculitis-related cardiomyopathy, coronary artery involvement, myocarditis, and myocardial ischemia. In a study by Schrader et al., 50% of autopsied PAN patients showed signs of coronary arteritis [62]. Coronary artery aneurysms are another cardiac manifestation of PAN [63]. The sites most commonly associated with coronary artery aneurysms are the proximal and central segments of the right coronary artery (RCA), followed by the initial segments of the left anterior descending (LAD) and left circumflex (LCX) arteries [64]. Although the most common coronary angiographic finding in polyarteritis nodosa (PAN) is the development of multiple aneurysms, there have been few isolated cases of coronary dissection [65]. Although it is a very rare presenting symptom, up to 30% of PAN patients have been documented to have acute myocardial infarction [66]. Since invasive angiography has far better diagnostic accuracy than non-invasive angiography, it should be performed on the coronary, renal, and mesenteric arteries when PAN is suspected [66]. Prednisone in combination with cyclophosphamide is currently the recommended therapeutic approach for PAN [64].

Granulomatosis with polyangiitis

Granulomatosis with polyangiitis is a granulomatous necrotizing vasculitis involving small to medium vessels, with a pronounced predilection for the upper and lower respiratory system and kidneys [67]. It is characterized by the presence of anti-neutrophil cytoplasmic antibodies (ANCA) [68]. The pathogenesis of this disorder encompasses a complex interplay between genetic, immunological, environmental, and infectious factors [69].

Cardiac involvement is relatively uncommon compared to other forms of vasculitis, with an incidence rate of 3.3% in a North American cohort [70], which includes pericarditis, coronary vasculitis, coronary artery aneurysms, myocarditis, endocarditis, valvular lesions, and conduction system abnormalities [71]. Pericarditis and pericardial effusion are the most frequent cardiac complications seen in GPA patients [72]. Ramirez et al. reported pericardial effusion in a GPA patient without uremia suggesting an autoimmune linkage of the condition [73]. A transthoracic echocardiogram could be performed in suspected cases. Most patients with GPA who present with pericarditis and associated effusion respond well to steroids. Imazio et al. found that steroid-unresponsive patients can be managed with a novel drug Anakinra which is deemed safe and effective [74].

The presence of a coronary artery aneurysm was initially reported by Rehani and Nelson in a nine-year-old girl with GPA [75]. Similar case reports in pediatric, adolescent, and adult patient populations were reported later [76,77]. Cardiac MRI is an effective technique for the early identification of cardiac abnormalities in patients diagnosed with GPA [78].

According to a retrospective cohort analysis by Kim et al. [79], GPA significantly increased the risk of heart failure. The incidence rate of heart failure was notably higher among individuals affected by GPA compared to those without the condition (RR: 7.2; 95% CI 6.4-8.1).

Valvular abnormalities can occasionally be caused by granulomatosis with polyangiitis, with the aortic valve being the most commonly affected [80]. Arrhythmias associated with GPA, albeit rare, include atrial tachycardia, fibrillation, and flutter [81]. A few cases of atrioventricular block have also been noted [82,83]. Prompt diagnosis using electrocardiography and management with telemetry monitoring, and sometimes a pacemaker, may be required.

Patients with GPA are commonly managed with immunosuppressants [68]. The predominant therapies for the induction phase are cyclophosphamide or rituximab, in conjunction with corticosteroids. The maintenance phase can be managed with methotrexate, azathioprine, or rituximab [68].

Eosinophilic granulomatosis with polyangiitis 

Eosinophilic granulomatosis with polyangiitis (EGPA), also referred to as Churg-Strauss syndrome, is an inflammatory vascular condition that affects both small- and medium-sized blood vessels, along with several organs. Around 27% to 47% of cases exhibit symptomatic cardiac involvement, with a higher prevalence among the ANCA-negative population [84].

Cardiac involvement is evident during the initial phase of the disease and is strongly associated with eosinophilia [85]. Patients can develop myocarditis, heart failure, pericarditis, tamponade, and myocardial infarction, the majority of which are associated with a poor prognosis [86]. Eosinophilic myocarditis (also known as Loeffler myocarditis) is the most common presentation, which leads to possible left ventricular dysfunction, aneurysms, and mural thrombi [87].

Myocarditis leads to persistent impairment of the left ventricular function, eventually causing either restrictive or dilated cardiomyopathy [87]. Neumann et al. evaluated 49 patients with EGPA, among whom 22 patients (45%) had cardiac disease, and endomyocarditis was the main presentation in 13 of these patients, confirmed by CMR or histopathologic examination of an endomyocardial biopsy [85]. Approximately 15-20% of EGPA patients with cardiovascular involvement develop acute pericarditis, potentially complicated by sizeable pericardial effusion and tamponade [87]. Involvement of the coronary vessels is frequently associated with myocardial infarction and heart failure and could result in coronary spasms manifesting as vasospastic angina [87].

Owing to its high reliability, CMR is the foremost diagnostic modality for assessing early cardiovascular complications in individuals diagnosed with EGPA [88]. Timely treatment and control of these cardiovascular complications are of immense importance, as they not only help alleviate symptoms but also facilitate achieving an early state of remission. Therefore, prompt and intensive therapy involving corticosteroids and cyclophosphamide has consistently demonstrated significant enhancements in cardiac outcomes and overall prognosis in individuals diagnosed with EGPA [88].

Microscopic polyangiitis

Microscopic polyangiitis (MPA) is a small vessel vasculitis that primarily affects the kidneys, lungs, and skin but can also involve other organ systems, such as the nervous system or gastrointestinal tract [89]. Most MPA patients have antibodies directed against myeloperoxidase (MPO-ANCA), but some may have antibodies against proteinase 3 (PR3-ANCA) [90]. It has been proposed that genetic, environmental, and microbial agents as well as therapeutic drugs may contribute to its etiopathogenesis [91].

Cardiovascular manifestations of MPA are very rare. Pericarditis and pericardial effusion/tamponade, valvular abnormalities, myocarditis, left atrial (LA) dilation, LV diastolic dysfunction, and arrhythmia are reported to be seen in patients with MPA. In a prospective study conducted on 132 patients with ANCA-positive microscopic polyangiitis, researchers found that cardiac complications were prevalent among the participants. Out of these patients, it was observed that 26 individuals experienced some form of cardiac damage, while 12 had pericarditis or pericardial effusion. Additionally, nine individuals suffered from chronic heart failure or cardiomyopathy, and there was one case each of aortic insufficiency and new-onset arrhythmia [92].

In the literature, only a few case reports of pericarditis and pericardial effusion/tamponade are published [93,94]. In a study by Thompson et al. [94], 267 of 1058 patients had microscopic polyangiitis out of which 27 patients had manifestations of pleuritis and/or pericarditis. These patients underwent echocardiography or computerized tomography (CT) chest for a definitive diagnosis of pericarditis and were either treated symptomatically or with immunosuppressive medications like glucocorticoids and cyclophosphamide or with pericardiocentesis depending upon the severity [94].

A case report by Shah et al. [95] emphasized prompt diagnosis and management with immunosuppressive medications like steroids and rituximab in a case of cardiac tamponade in an MPA patient. Kim et al. described a rare case of severe acute aortic valve insufficiency in an MPA patient [96]. Despite intensive treatments, the 56-year-old patient suffered sudden cardiac arrest and died.

A 77-year-old Caucasian female was diagnosed with a complete heart block caused by MPA, according to a case report published by Filice et al. [97]. It was attributed to coronary artery necrosis due to vasculitis. The cornerstone of the treatment of MPA includes glucocorticoids and immunosuppressants such as cyclophosphamide, rituximab, and methotrexate [98].

Behcet disease 

Behcet disease (BD) is an autoimmune vasculitis with a multi-systemic spread, commonly involving the skin with recurrent painful mucocutaneous ulcerations constituting the clinical hallmark, along with ocular, neurologic, and cardiac lesions. Although unclear, the close affiliation of cases with HLA-B51 along with some bacteria and viruses indicate that genetic, environmental, and infectious triggers play a role in the activation of T-cell mediated immune response (Th1 & Th17) [99].

The prevalence of cardiovascular association is between 7% and 46%, with venous involvement (29%) being more common than arterial (8-18%) involvement [100]. The most often reported vascular manifestations were superficial vein thrombosis and deep vein thrombosis, both of which were shown to be related to the right heart chambers as well as the vena cava and pulmonary veins [101]. Geri et al. studied 807 cases and found a 6% prevalence of cardiovascular association, among which pericarditis (38%), endocarditis (26%), intracardiac thrombus (19%), and myocardial infarcts (17%) were among the most common manifestations [102]. Yavne et al. also found a significantly higher fraction of ischemic heart disease among BD cases compared to matched controls [103].

Electromechanical conduction delay, along with QRS abnormalities have also been noted [104]. Coronary aneurysms, though rare, can manifest as acute coronary syndrome [105]. In a retrospective study by Vural et al., patients with BD were found to have a higher prevalence of angina (31%), acute coronary syndrome (8%), and arrhythmia (8%), with 15% of patients with coronary obstruction showing early mortality to acute myocardial infarction [106]. According to Aslam et al.'s review, patients with BD had a statistically significant decline in ejection fraction when compared to controls [107]. Significant improvements in the ejection fraction and ventricular function were noted in the BD group who received high-dose prednisone and azathioprine therapy in addition to the standard cardiac failure treatment [108]. Pericardial involvement is a frequently reported cardiac manifestation of Behcet’s disease [109].

Echocardiography serves as a valuable non-invasive diagnostic method for identifying cardiac abnormalities in BD [110]. The use of glucocorticoids has been linked to an increased risk of cardiovascular mortality [111]. Cyclophosphamide holds to be the first-line agent against critical vascular events with maintenance therapy using mycophenolate mofetil as well as azathioprine shown to reduce mortality, with the latter being strictly recommended in patients with BD [112].

## Conclusions

Vasculitides are autoimmune disorders that target various parts of the vasculature in the body. Emerging evidence suggests that primary systemic vasculitides are becoming increasingly recognized as a prominent contributor to cardiovascular morbidity and mortality among younger individuals. Among these associations, accelerated atherosclerosis, ischemic heart disease, venous thromboembolism, involvement of the aorta, valvular disorders, and inflammation/structural changes in the myo-pericardial region were frequently observed. It is imperative for healthcare professionals to recognize and monitor these potential complications carefully. Patients with systemic vasculitides, especially those with long-standing disease or risk factors, should be recommended to undergo regular screening using non-invasive imaging techniques, inflammatory markers, and electrocardiograms (EKG). This comprehensive approach allows physicians to assess the extent of cardiovascular involvement and monitor any potential complications associated with vasculitis. Treatment for these conditions typically involves the use of glucocorticoids and immunomodulators. However, healthcare providers must carefully evaluate the potential risks versus benefits when determining therapy options for each specific type of vasculitis and its impact on cardiovascular health. 

Clinicians should regularly assess patients' cardiovascular status using appropriate screening methods and tailor therapeutic approaches accordingly to optimize outcomes. Through this approach, clinicians aim to minimize damage caused by systemic inflammation and reduce the likelihood of developing complications related to atherosclerosis.
